# Effects of replacing soybean oil with palm oil on growth performance, appetite, and gut health in weaned piglets

**DOI:** 10.3389/fvets.2025.1682514

**Published:** 2026-02-12

**Authors:** Dan Li, Kun Wu, Hanxin Liu, De Wu, Lianqiang Che, Zhengfeng Fang, Bin Feng, Shengyu Xu, Yong Zhuo, Lun Hua, Josh Wen-Cheng Chiu, Guangmang Liu, Yan Lin

**Affiliations:** 1 Key Laboratory of Animal Disease-Resistance Nutrition and Feed Science, Institute of Animal Nutrition, Sichuan Agricultural University, Chengdu, Sichuan, China; 2 College of Life Sciences, Sichuan Agricultural University, Yanan, Sichuan, China; 3 Ecolex Animal Nutrition, Kuala Lumpur, Malaysia

**Keywords:** appetite, digestibility, growth performance, palm oil, piglets

## Abstract

**Introduction:**

Weaned piglets face growth challenges due to low feed intake and weaning stress. Palm oil (PO) rich in long-chain saturated fatty acids may optimize nutrition, but appropriate replacement ratios and mechanisms remain unclear.

**Methods:**

144 weaned piglets were randomly assigned to four groups (CON: 4% soybean oil (SO); T1-T3: 25%, 50%, 100% SO replaced by PO) for 35 days. Growth performance, nutrient digestibility, intestinal health, gene expression, and gut microbiota were measured.

**Results:**

T1 and T3 improved average daily gain (ADG) and reduced feed/gain ratio (0-14 d, *p*<0.05); T3 increased ADG (0-28 d) and average daily feed intake (ADFI, *p*<0.05). PO reduced ether extract digestibility (*p*<0.05), upregulated ZO-1/Claudin-1 mRNA (T3, *p*<0.05), and increased hypothalamic NPY/AGRP expression. Gut microbial diversity (OTU abundance) and beneficial bacteria (*Roseburia*) were enhanced in T3.

**Discussion:**

Moderate PO replacement (25%) balances growth promotion and digestibility, while high replacement (100%) improves gut barrier function and appetite. PO regulates growth via fatty acid composition, appetite-related genes, and gut microbiota

## 1 Introduction

When piglets are weaned, the digestive system of piglets is underdeveloped, and changes associated with weaning, such as psychological stress (1), nutritional shifts (2), and environmental factors (3), can easily lead to diarrhea, reduced appetite, poor feed efficiency, growth suppression, and even death, collectively known as “weaning stress syndrome” in piglets (4). Among these issues, low feed intake early after weaning and insufficient energy are significant factors that hinder piglet growth. Therefore, improving feed intake and promoting growth performance in weaned piglets remains a key focus of research and attention in the pig farming industry.

Maternal milk, an important source of nutrition for piglets, provides essential energy for growth. Most fatty acids in milk fat are long-chain fatty acids. Milk fat is rich in palmitic acid, accounting for 34.23% of its total fatty acid composition, with an unsaturated-to-saturated (U/S) fatty acid ratio of approximately 1.2–1.5 (5). Studies show that long-chain fatty acids can reduce energy expenditure and promote weight gain (6, 7). Some research also suggests that they can regulate CCK levels, decrease feelings of fullness, and increase average daily food intake (ADFI) (8). Additionally, long-chain saturated fatty acids can enhance the energy content of the diet (9). Palm oil (PO), which contains palmitic acid and is rich in long-chain fatty acids, has been investigated as a substitute for soybean oil (SO) in weaned piglet diets. Replacement of SO with PO in weaned piglet diet does not negatively affect performance (e.g., average daily gain (ADG), ADFI, feed to gain ratio (F/G)), but it does improve piglet health and reduce mortality by 25–55% (10). Adding 6% PO to the diets of weaned piglets significantly increased their ADG and ADFI in the PO group compared with piglets fed 6% SO (11). However, few studies have examined appropriate ratios of PO and SO or the physiological mechanisms regulating feed intake in piglets.

Although SO is widely used in production due to its high C18:2 content, it has lower C16:0 content than breastmilk. To better match the fatty acid composition of maternal milk and provide sufficient energy for piglets by adjusting diet components, it is important to balance the dietary fatty acid composition. This balance may enhance feed intake, promote growth performance, and improve intestinal microflora. Therefore, we investigate the effect of replacing SO with PO on the growth performance of weaned piglets and its potential underlying mechanisms.

## 2 Materials and methods

### 2.1 Ethics statement

In this study, the experiment was conducted in accordance with relevant animal protection laws and approved by the Institutional Animal Care and Use Committee of Sichuan Agricultural University (Approval No.: SCAUAC202308-4).

### 2.2 Experimental design and animal diets

In this experiment, 144 weaned piglets, 21 days of age with balanced sex (7.65 ± 0.04 kg), were selected and randomly divided into four treatments, with 12 replicates and three piglets/replicate. The control group (CON) was fed a basal diet (4% SO, CON), with SO replaced by PO at 25% (3%SO+1%PO, T1), 50% (2%SO+2%PO, T2), and 100% (4% PO, T3) for 35 days. The proportion of oils and composition of fatty acids in the experimental diets are presented in Table 1. The PO was supplied by Ecolex Animal Nutrition, Kuala Lumpur, Malaysia. Diets fed to weaned piglets were allocated according to the nutritional requirements of 7–11 kg and 11–25 kg piglets in the NRC (12). The dietary formula and nutritional levels are shown in Supplementary Tables 1, 2. On Day 28 of the experiment, eight healthy weaned piglets per treatment (body weight closest to group mean) were selected for a 7-day digestibility experiment, with one pig per replicate. The experimental diets contained an additional 0.3% chromium oxide (Cr₂O₃) as an indigestible marker.

**Table 1:** Composition of fatty acids in the experimental diet.

Item	CON	T1	T2	T3
Replacement ratio, %	-	25%	50%	100%
Proportion of mixed oil, %				
Soybean oil, %	4	3	2	0
Palm oil^1^, %	0	1	2	4
Fatty acids, % of total fatty acids^2^				
C16:0	11.76	19.92	30.12	41.78
C18:0	3.85	3.97	4.04	3.90
C18:1	21.76	20.79	19.60	18.07
C18:2	48.28	41.83	33.90	25.05
LCFA	95.10	95.08	95.01	94.97
MCFA	0.08	0.06	0.08	0.08
USFA	78.14	69.85	59.44	47.64
SFA	17.06	25.31	35.68	47.41
U/S	4.58	2.76	1.67	1.00
n-3	7.38	6.58	5.33	3.94
n-6	48.45	41.97	33.99	25.08
n-6/n-3	6.57	6.38	6.38	6.36

LCFA: long-chain-fatty-acid, C > 12; MCFA: Midchain-fatty-acids, C = 6–12.

1 Palm oil: With a fat content of 99%, it provides 69–74% C16:0, 4–6% C18:0, 14–18% C18:1, and 14–18% C18:2.

2 Fatty acid content in the diet was the measured value.

### 2.3 Management

Piglets were housed in fully-slatted pens (1.8 m × 1.0 m). Environmental conditions included an ambient temperature of 28 °C, 65–75% relative humidity, and twice-weekly disinfection. At 0–28 d, piglets were fed four times a day (0800, 1200, 1600, and 2000 h); at 29–35 d, piglets were fed three times per day (0800, 1400, and 2000 h) and had free access to both feed and water. Dietary transition followed a 3-day graded protocol (2:1 → 1:2 → 100% new diet).

### 2.4 Performance measurement and sample collection

Piglets were individually weighed on days 0, 14, and 28. During the experimental period, the daily FI was weighed at 0700 h each morning based on the previous day’s FI, and the remaining feed in both feeders and troughs was weighed at 2130 h each evening to calculate daily FI. Data were used to calculate the ADG, ADFI, and F/G.

Fecal consistency was categorized daily as 0, normal; 1, pasty; 2, semi-liquid; or 3, liquid (13). The occurrence of diarrhea was defined as fecal scores ≥ 2 (14). Fecal scores of piglets in each litter were recorded daily, and their diarrhea rate (DR) and diarrhea index (DI) were calculated (15).

Blood samples were collected via anterior vena cava puncture on days 14 and 28, blood was centrifuged for 10 min at 3000 rpm, and the serum was stored at −80 °C.

Feed samples were collected after each mixing event using the quartering method. Representative samples were sealed in zip-lock bags and stored at −20 °C for subsequent analysis. On days 14 and 28, 8 piglets were selected from each treatment group, and fecal samples were collected by stimulating defecation with sterile cotton swabs rubbed against the piglets’ anus. The samples were stored in sterile PC tubes at −80 °C for 16S rDNA gene sequencing analysis. On days 33–35, 8 piglets were selected from each treatment group. Fecal collection began when the piglets’ feces turned completely green. Six piglets were randomly selected from each treatment group, and fecal samples were collected (5 mL of 10% sulfuric acid per 100 g of feces) for three consecutive days and stored at −20 °C.

On day 28, 6 piglets were selected from each treatment group. After euthanasia (Zoletil 50® Virbac, France, 10 mg/kg), the cranial cavity was opened to extract the hypothalamus, which was then minced. The middle segment of the jejunum was excised and rinsed with physiological saline, and the mucosal layer was scraped using a glass slide. All samples were aliquoted into 1.5 mL sterile EP tubes, wrapped in aluminum foil, flash-frozen in liquid nitrogen, and stored at −80 °C for further analysis.

### 2.5 Analyses

#### 2.5.1 Biochemical indicator of blood

Alanine aminotransferase (ALT), aspartate aminotransferase (AST), alkaline phosphatase (ALP), total protein (TP), albumin (ALB), glucose (GLU), total bile acid (TBA), nonestesterified fatty acid (NEFA), total cholesterol (TC), low-density lipoprotein-cholesterol (LDL-C), triglyceride (TG), and high-density lipoprotein-cholesterol (HDL-C) contents in piglet serum were measured by the Hitachi 3,100 Automatic Analyzer (Hitachi High-Tech (Shanghai) Co., Ltd., China).

#### 2.5.2 Determination of jejunal mucosal morphology

A 1-cm jejunal segment was collected from the same middle-upper position in each piglet and fixed in 4% paraformaldehyde solution. After fixation, embedding, and sectioning, the jejunal tissues were stained with hematoxylin and eosin (H&E) for morphological examination. Villus height and crypt depth were measured using an OLYMPUS BX43 microscope (Japan).

#### 2.5.3 Apparent total tract digestibility (ATTD) of nutrients

Gross energy (GE), crude protein (CP), ether extract (EE), neutral detergent fiber (NDF), and Cr levels in the feed and fecal samples were analyzed (16). GE contents were analyzed using an adiabatic oxygen bomb calorimeter (Parr 6,400 Instruments Co., Moline, IL, USA). EE and Cr contents were analyzed (16). The CP was calculated using the Kjeldahl method. Total nitrogen was determined with a Kjeltec analyzer (OLBA9870A, Jinan Olibo Scientific Instrument Co., Ltd., China). The content of CP = 6.25 × Total N. CF and NDF levels were analyzed using a fiber analyzer (Ankom Technology, Macedon, NY, USA) (17). The apparent total tract digestibility (ATTD) of each nutrient was calculated according to the relevant requirements of the endogenous indicator method (18).


ATTD=[1−(a÷b)×(c÷d)]×100%

Where *b* is the content of a nutrient in the diet; *a* is the content of a nutrient in the fecal sample; *c* is the content of the indicator (Cr) in the diet; and *d* is the content of the indicator (Cr) in the fecal sample.

#### 2.5.4 Long-chain fatty acid (LCFA) analysis

LCFA were analyzed according to AOAC Official Method 996.06 (19) with modifications. 1 g of prepared feed samples was added to 3 mL of a chloroform-methanol–water solution (volume ratio 8:4:3), then vortexed for 1 min, and then sonicated in an ice bath for 5 min. Repeat 3–5 times, and let it stand for 2 h. The solution was then centrifuged at 1500 rpm for 5 min; the chloroform (bottom) layer was collected, washed twice with a chloroform-methanol–water mixture, and dried in a vacuum drying oven to obtain fatty acid glycerides. To these glycerides, 2 mL of KOH-CH3OH solution (c = 0.5 mol/L) was added in a reaction tube, which was then vortexed for 1 min and placed in a 50 °C water bath for 10 min to complete saponification (until oil droplets had disappeared), yielding the free fatty acid mixture. After cooling, 2 mL of BF3-CH3OH solution (w = 14%) was added to the free fatty acid mixture, which was then vortexed for 10 s, then transferred to an 80 °C water bath and heated under reflux for 2 min to complete methylation. The sample was then cooled to room temperature before 1 mL of n-hexane (0.05 g/L BHT n-hexane solution) and 2 mL of saturated NaCl solution were added, and the solution was vortexed thoroughly for extraction, then centrifuged at 1000 rpm for 5 min at room temperature; the upper n-hexane layer (≥ 600 μL) was collected for testing. Sample fatty acid composition was analyzed using a Shimadzu GC-2010 gas chromatograph (Shimadzu Scientific Instruments, Columbia, MD).

#### 2.5.5 16S rRNA sequencing and data analysis

The number of microorganisms in piglet feces was determined using 16S rRNA. Total microbial genomic DNA was extracted from fecal samples using the FastPure Stool DNA Isolation Kit (MJYH, Shanghai, China) following the manufacturer’s instructions. DNA quality and concentration were determined by 1.0% agarose gel electrophoresis and a NanoDrop® ND-2000 spectrophotometer (Thermo Scientific Inc., USA). The hypervariable region V3-V4 of the bacterial 16S rRNA gene was amplified using primer pairs 338F (5’-ACTCCTACGGGAGGCAGCAG-3′) and 806R (5’-GGACTACHVGGGTWTCTAAT-3′) by a T100 Thermal Cycler (BIO-RAD, USA). PCR amplification cycling conditions were as follows: initial denaturation at 95 °C for 3 min, then 27 cycles of denaturing at 95 °C for 30 s, annealing at 55 °C for 30 s, an extension at 72 °C for 45 s, and a single extension at 72 °C for 10 min, and stored at 4 °C. PCR product was extracted from 2% agarose gel, purified, then quantified using Synergy HTX (Biotek, USA). Purified amplicons were pooled in equimolar amounts and paired-end sequenced on an Illumina NextSeq 2000 PE300 platform (Illumina, San Diego, USA) following standard protocol from Majorbio Bio-Pharm Technology Co., Ltd. (Shanghai, China).

#### 2.5.6 RNA extraction and quantitative real-time PCR (qRT-PCR)

Relative mRNA expression of genes involved in feeding regulation was determined by quantitative real-time PCR (qRT-PCR). Total RNA was extracted from frozen hypothalamic samples using TRIzol (Invitrogen, Carlsbad, CA, USA). RNA concentration was measured using a nucleic acid protein detector (DU800, Focus Technology). A 1 μg sample of isolated total RNA was synthesized using a reverse transcription kit (HiScript III RT SuperMix for qPCR (+ gDNA wiper), Nanjing Vazyme Biotech Co., Ltd., China) following manufacturer instructions. qRT-PCR analysis was performed on a QuantStudio 5 real-time PCR system (Thermo Fisher Scientific, USA) using a fluorescence quantification kit (ChamQ Universal SYBR qPCR Master Mix, Nanjing Vazyme Biotech Co., Ltd., China). The PCR protocol involved 1 cycle of 30 s at 95 °C, followed by 40 cycles of 10 s at 95 °C and 30 s at 60 °C. The last cycle continued for 15 s at 95 °C, 1 min at 60 °C, and 15 s at 95 °C. qRT-PCR data were analyzed using the 2-delta CT method with *β*-actin as a reference. Primer base sequences for the genes are presented in Table 2.

**Table 2:** Primer sequences of the target and reference genes.

Genes	Primer	Sequence (5′ → 3′)	Accession no.
β-actin	Forward	GGATGCAGAAGGAGATCACG	XM_021086047.1
	Reverse	ATCTGCTGGAAGGTGGACAG	
	Reverse	CCCAGTTGTCCAGGAGTTTCAG	
NPY	Forward	TCACCAGGCAGAGATACGGA	NM_001256367.1
	Reverse	ACACAGAAGGGTCTTCGAGC	
POMC	Forward	GTGGCTGGTGCTTGGAAAG	NM_213858.1
	Reverse	GAAGTGGCCCATGACGTACT	
PYY	Forward	GGAGGAGCTGAGCCGCTACTAC	NM_001256528.1
	Reverse	GCTGTCACGTTTCCCATACCTCTG	
AGRP	Forward	AGAGGACAACTGCAGAACGG	NM_001011693.1
	Reverse	AGGATCTAGCACCTCTGCCA	
5-HT	Forward	ATGCAGTCCATCAGCAACGA	NM_214217.1
	Reverse	ATGACGGCCATGATGTTGGT	
CCK	Forward	CAGGCTCGAAAAGCACCTTC	NM_214237.2
	Reverse	GCGGGGTCTTCTAGGAGGTA	
Occludin	Forward	ATGCTTTCTCAGCCAGCGTA	NM_001163647.1
	Reverse	AAGGTTCCATAGCCTCGGTC	
ZO-1	Forward	GAGGATGGTCACACCGTGGT	XM_003353439.1
	Reverse	GGAGGATGCTGTTGTCTCGG	
Claudin-1	Forward	AAGGACAAAACCGTGTGGGA	NM_001244539.1
	Reverse	CTCTCCCCACATTCGAGATGATT	
NF-κB	Forward	AGCCATTGACGTGATCCAGG	NM_001048232.1
	Reverse	CGAAATCGTGGGGCACTTTG	
mTOR	Forward	CATTGGAGATGGTTTGGTGA	XM_003127584.4
	Reverse	ATGGGATGTGGCTTGTTTGA	

NPY, Neuropeptide Y; POMC, Pro-opiomelanocortin; PYY, Peptide YY; AGRP, Agouti-related protein; 5-HT, 5-hydroxytryptamine.

### 2.6 Statistical analysis

Statistical analysis was performed using SPSS 21.0 software (IBM SPSS Company, Chicago, Illinois, USA). Analysis included one-way ANOVA, followed by Tukey’s multiple comparisons. Normality was assessed by the Shapiro–Wilk test, and homogeneity of variances was assessed using the Levene test. Results are presented as means and standard error of the mean (SEM). Differences were considered significant at *p* < 0.05 and extremely significant at *p* < 0.01. Bioinformatic analysis of gut microbiota was performed using the Majorbio Cloud platform. Based on ASVs information, alpha diversity indices, including ACE and Chao indices, were calculated with Mothur v1.30.2. Similarity among microbial communities in samples was determined by principal coordinate analysis (PCoA) based on Bray–Curtis dissimilarity using the Vegan v2.4.3 package. The correlation coefficients between ADFI, BW, ADG, and the 25 dominant bacterial species were determined using Pearson correlation analysis, with the resulting numerical matrix visualized as a heatmap.

## 3 Results

### 3.1 Effect of palm oil replacement for soybean oil on growth performance

As shown in Table 3, the T1 and T3 groups showed significant improvements in BW on day 14 and ADG from 0–14 days. The T3 group increased BW at 28 d, ADG from 0–28 d, and ADFI throughout the experiment (*p* < 0.05). Replacing SO with PO decreased F/G from 0–14 d (*p* < 0.05). As shown in Table 4, replacing SO with PO showed no significant difference in the DR of weaned piglets, but T1 and T3 showed a trend toward decreased DI from 0–28 d (*p* = 0.08).

**Table 3:** Effect of palm oil replacement for soybean oil on growth performance.

Item	CON	T1	T2	T3	SEM	*p*-value
BW, kg						
0 d	7.66	7.67	7.65	7.65	0.04	0.10
14 d	10.29^a^	11.31^b^	10.69^a^	11.37^b^	0.11	< 0.01
28 d	17.12^a^	17.84^ab^	16.98^a^	18.57^b^	0.19	0.01
0–14 d						
ADG, g	196.8^a^	259.72^b^	210.63^a^	265.98^b^	7.10	< 0.01
ADFI, g	390.25^a^	412.69^a^	377.18^a^	461.98^b^	9.05	< 0.01
F/G	2.01^c^	1.6^a^	1.81^b^	1.76^ab^	0.04	< 0.01
DR, %	0.50	0.39	0.54	0.36	0.05	0.59
DI	0.14	0.10	0.13	0.08	0.01	0.28
15–28 d						
ADG, g	479.29	465.07	453.33	513.98	10.41	0.16
ADFI, g	739.69^ab^	778.52^b^	705.37^a^	871.71^c^	14.41	< 0.01
F/G	1.58	1.68	1.57	1.71	0.03	0.18
DR, %	0.67	0.53	0.63	0.36	0.07	0.41
DI	0.17	0.15	0.15	0.07	0.02	0.14
0–28 d						
ADG, g	338.04^a^	363.1^ab^	333.07^a^	389.98^b^	12.65	0.01
ADFI, g	564.97^a^	590.62^a^	558.05^a^	666.85^b^	10.54	< 0.01
F/G	1.69	1.63	1.68	1.71	0.02	0.43
DR, %	0.60	0.46	0.70	0.32	0.06	0.11
DI	0.15	0.13	0.17	0.08	0.01	0.08

^a,b^ Values in the same row with different small letter superscripts mean significant difference (*p* < 0.05). Data are presented as mean (*n* = 12). Data are presented as means and standard error of the mean (SEM).

BW, Body weight; ADG, Average daily gain; ADFI, Average daily feed intake; F/G, Feed/gain ratio; DR, diarrhea rate; DI, diarrhea index.

**Table 4:** Effect of palm oil replacement for soybean oil on serum biochemical parameters.

Item	CON	T1	T2	T3	SEM	*p*-value
14 d						
ALT, U/L	52.07	59.15	52	49.8	2.17	0.475
AST, U/L	43.68	44.47	49.94	42.25	2.06	0.739
ALT/AST	0.88	0.76	0.95	0.86	0.03	0.22
TP, g/L	42.22	40.33	42.06	40.8	0.46	0.40
ALB, g/L	21.11	21.02	21.26	20.64	0.37	0.95
ALP, U/L	237.4	249.5	234.67	223.83	9.81	0.85
TC, mmol/L	1.49	1.43	1.56	1.40	0.04	0.47
LDL-C, mmol/L	0.80	0.80	0.88	0.81	0.02	0.75
HDL-C, mmol/L	0.79	0.85	0.88	0.76	0.03	0.36
GLU, mmol/L	5.51	5.47	5.56	5.22	0.16	0.89
TBA, μmol/L	9.80	19.47	20.55	22.86	2.58	0.37
NEFA, μmol/L	72.05	32.74	54.73	46.98	5.34	0.07
TG, mmol/L	0.42	0.36	0.41	0.36	0.02	0.61
28 d						
ALT, U/L	58.61	57.63	54.56	41.11	3.01	0.14
AST, U/L	47.00	68.3	43.42	31.65	4.78	0.07
ALT/AST	0.84	1.17	0.82	0.77	0.07	0.08
TP, g/L	41.36	44.05	38.84	32.63	1.53	0.18
ALB, g/L	21.72	23.58	21.34	17.31	0.85	0.27
ALP, U/L	186.20^ab^	188.83^b^	172^ab^	125.67^a^	9.13	0.03
TC, mmol/L	1.45	1.73	1.40	1.18	0.09	0.19
LDL-C, mmol/L	0.82	1.02	0.76	0.7	0.05	0.15
HDL-C, mmol/L	0.82	0.85	0.76	0.58	0.04	0.21
GLU, mmol/L	5.09^ab^	5.89^b^	4.89^ab^	3.81^a^	0.24	0.01
TBA, μmol/L	33.12	23.83	16.42	36.81	3.60	0.19
NEFA, μmol/L	57.24	109.4	85.27	69.2	8.04	0.10
TG, mmol/L	0.32	0.37	0.32	0.3	0.02	0.64

^a,b^Values in the same row with different small letter superscripts mean significant difference (*p* < 0.05). Data are presented as mean (*n* = 6). Data are presented as means and standard error of the mean (SEM).

ALT, Alanine aminotransferase; AST, aspartate aminotransferase; TP, total protein; ALB, albumin; ALP, alkaline phosphatase; TC, total cholesterol; LDL-C, low-density lipoprotein-cholesterol; HDL-C, high-density lipoprotein-cholesterol; GLU, glucose; TBA, total bile acid; NEFA, nonestesterified fatty acid; TG, triglyceride.

### 3.2 Effect of palm oil replacement for soybean oil on serum biochemical indexes

Replacing SO with PO showed no significant difference in ALT, AST, TP, ALP, TC, LDL-C, HDL-C, GLU, and TG in weaned piglets on day 14 (Table 4). The T1 and T3 groups showed a trend toward a decrease in NEFA on day 14 (*p* = 0.07). On day 28, the T3 group had lower ALP and GLU levels than the T1 group (*p* < 0.05).

### 3.3 Effect of palm oil replacement for soybean oil on ATTD of nutrients in weaned piglets

Replacing SO with PO reduced ATTD of EE (*p* < 0.05) (Table 5). The ATTD of GE, CF, and NDF were significantly reduced in T1 and T3 (*p* < 0.05).

**Table 5:** Effect of palm oil replacement for soybean oil on nutrient digestibility in weaned piglets.

Digestibility	CON	T1	T2	T3	SEM	*p*-value
CP, %	83.39^b^	79.84^a^	85.42^b^	78.53^a^	0.70	< 0.01
GE, %	88.94^b^	86.45^a^	89.02^b^	86.21^a^	0.32	< 0.01
EE, %	86.41^d^	77.22^c^	70.78^b^	51.23^a^	2.38	< 0.01
CF, %	49.68^b^	34.14^a^	52.07^b^	28.60^a^	2.25	< 0.01
NDF, %	52.71^b^	45.20^a^	54.71^b^	46.05^a^	1.26	0.01

^a, b, c, d^ Values in the same row with different small letter superscripts mean significant difference (*p* < 0.05). Data are presented as mean (*n* = 8). Data are presented as means and standard error of the mean (SEM).

CP, Crude Protein; GE, Gross energy; EE, Ether extra; CF, Crude fiber; NDF, Neutral detergent fiber.

### 3.4 Effects of replacing soybean oil with palm oil on gut health in weaned piglets

**Figure 1:**
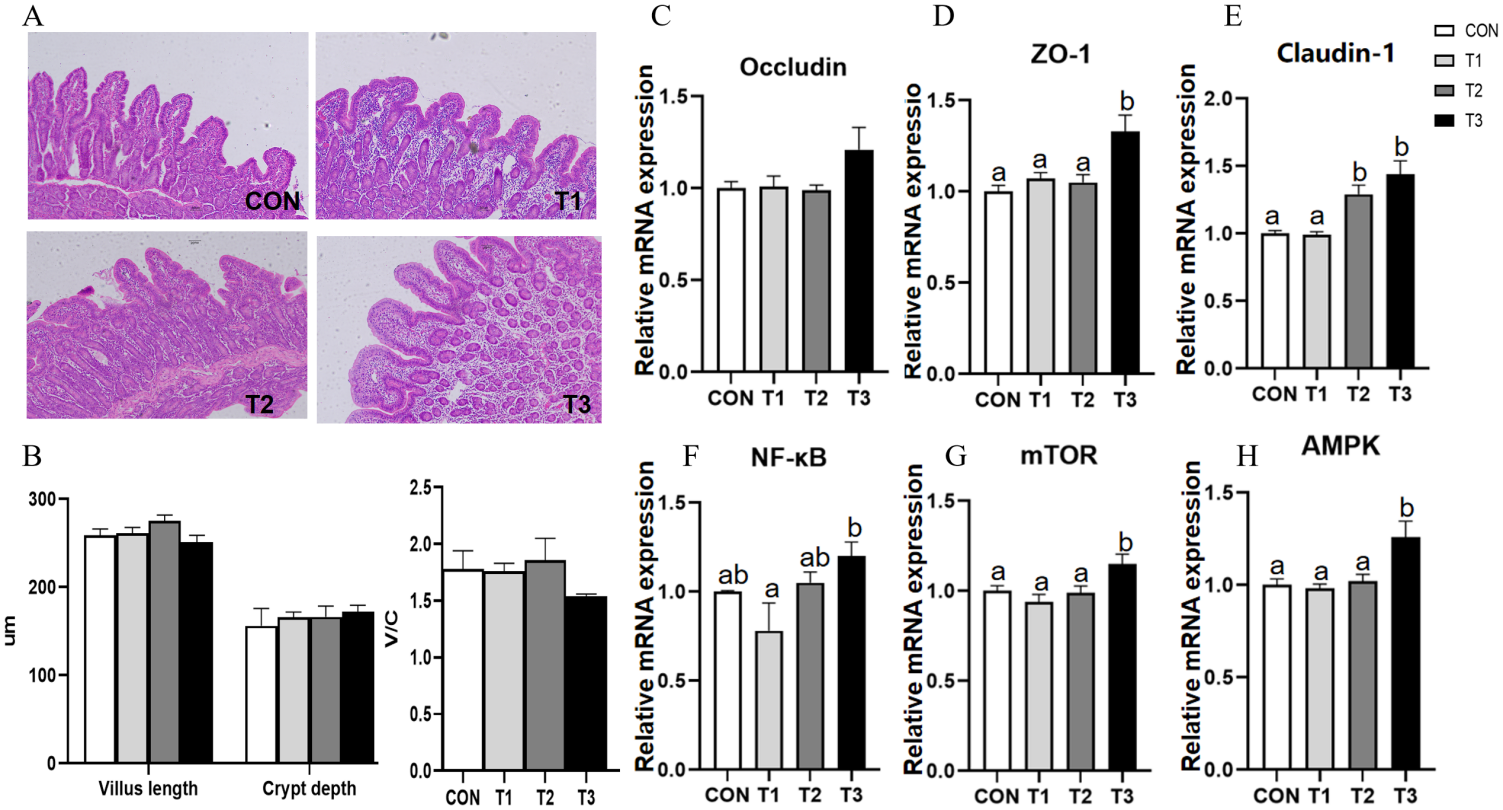
Effects of replacing soybean oil with palm oil on gut health in weaned piglets. **(A)** Jejunal morphology; **(B)** Villus height, crypt depth, and V/C ratio in the jejunum; **(C–H)** Relative mRNA expression of genes in jejunal mucosa. Different superscript letters indicate a significant difference (*p* < 0.05). Data are presented as mean (*n* = 6). *ZO-1*:onula occludens-1*NF-κB*: uclear factor kappa-B*AMPK*: Adenosine 5′-monophosphate (AMP)-activated protein kinase*mTOR*: ammalian target of rapamycin. Data are presented as means and standard error of the mean (SEM).

Replacement of SO with PO did not significantly affect intestinal villus height, crypt depth, or V/C ratio (*p* > 0.05) (Figures 1A,B). However, compared with the CON group, the T2 group showed significantly increased relative mRNA expression of Claudin-1 in jejunal mucosa, while the T3 group exhibited significantly upregulated mRNA expression of ZO-1, Claudin-1, mTOR, and AMPK (*p* < 0.05) (Figures 1C–H).

### 3.5 Effect of palm oil replacement for soybean oil on the mRNA expression of feeding-regulated genes

**Figure 2:**
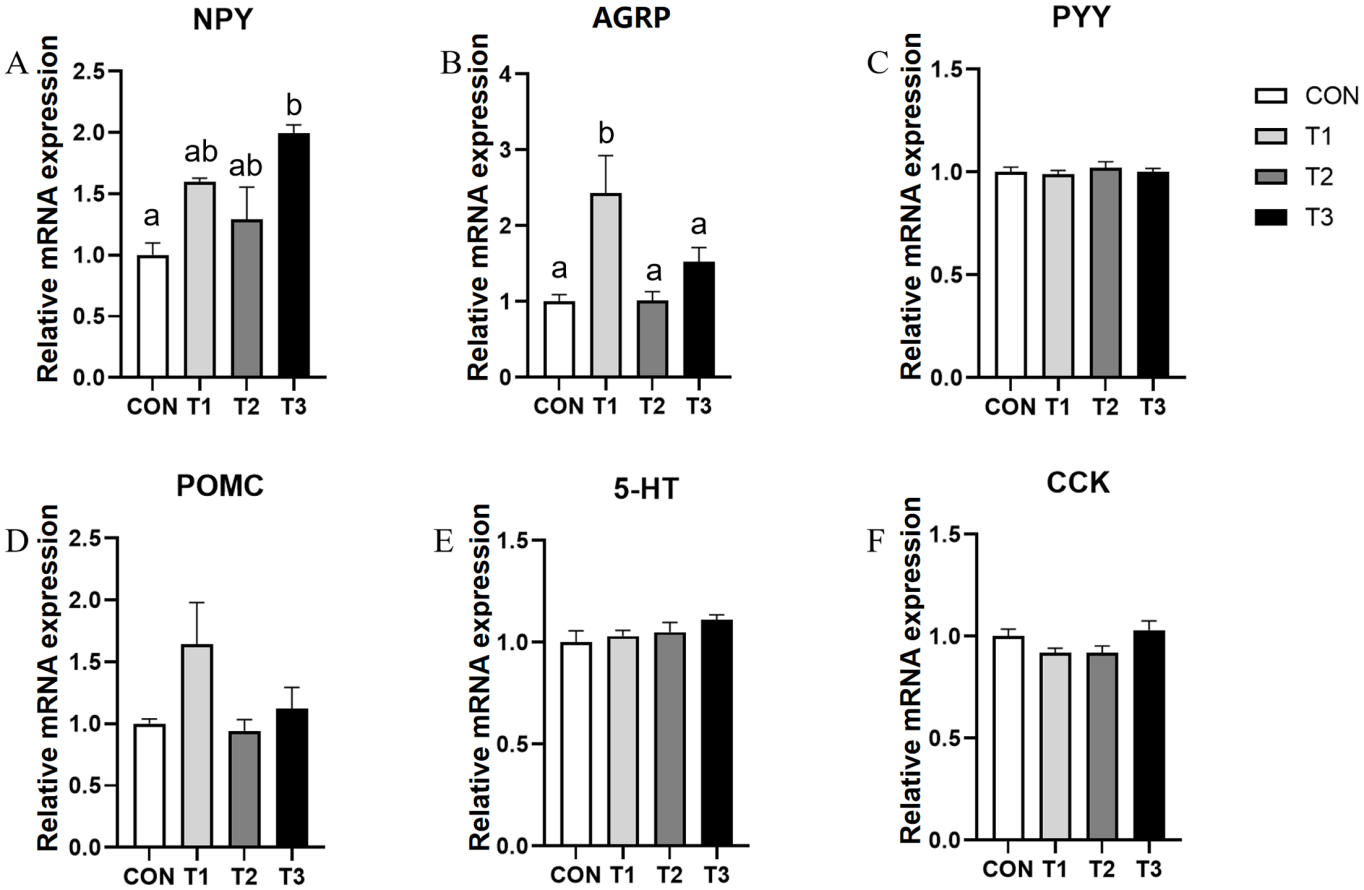
The mRNA levels of NPY **(A)**, AGRP **(B)**, PYY **(C)**, POMC **(D)**, 5-HT **(E)**, and CCK **(F)** in the hypothalamus of weaned piglets. Different superscript letters indicate a significant difference (*p* < 0.05). Data are presented as mean (*n* = 6). Data are presented as means and standard error of the mean (SEM). *NPY*, Neuropeptide Y; *POMC*, Pro-opiomelanocortin; *PYY*, eptide YY; *AGRP*, gouti-related protein; *5-HT*, 5-hydroxytryptamine.

The mRNA expressions of appetite regulatory genes in the hypothalamus are shown in Figure 2. The T3 group significantly increased the *NPY* mRNA expression, while the T1 group significantly increased the *AGRP* mRNA expression (*p* < 0.05). After replacing SO with PO, there were no significant differences in *PYY*, *POMC*, *5-HT*, or *CCK* mRNA expression.

### 3.6 Correlation analysis between dietary fatty acids and growth performance/nutrient digestibility in weaned piglets

**Figure 3:**
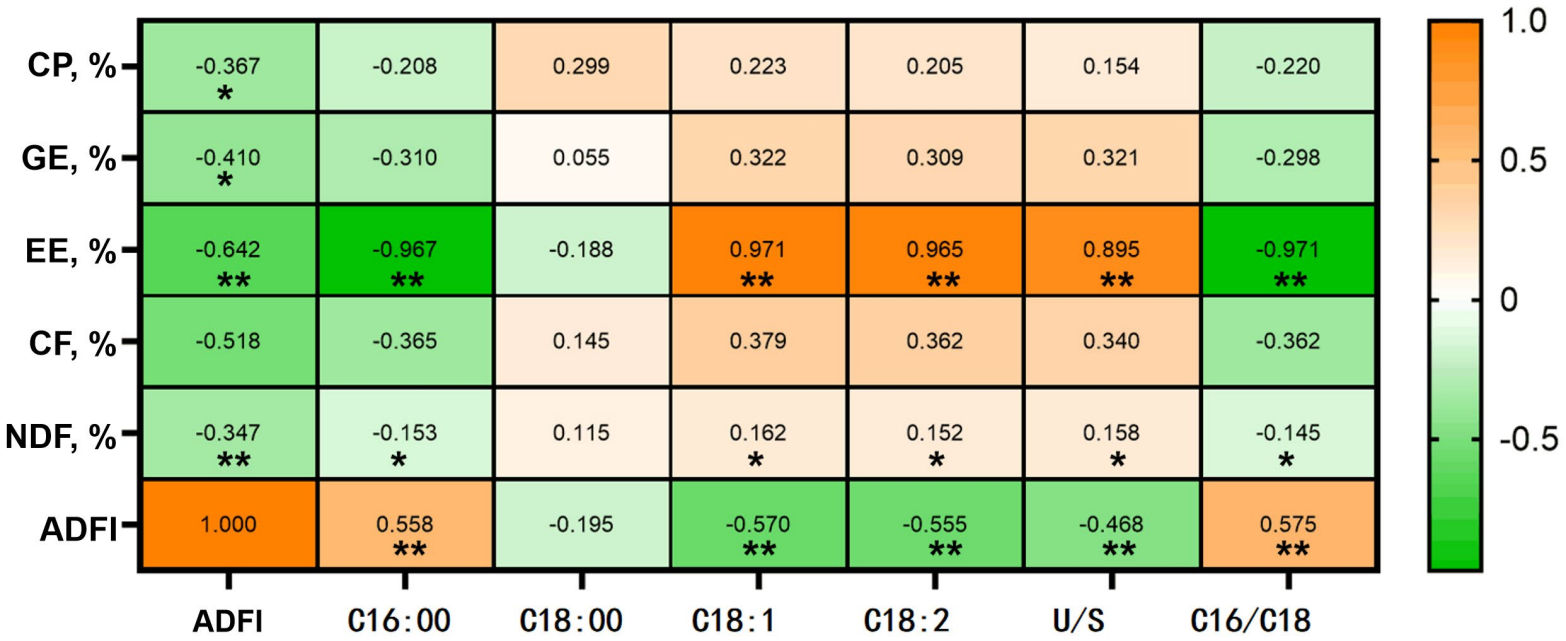
Correlation analysis between dietary fatty acids and growth performance/nutrient digestibility in weaned piglets. The color scale ranges from saturated yellow (1.0) to white (0) to saturated green (−1.0). Yellow and green colors represent increased and decreased expressions, respectively. *p < 0.05, **p < 0.01. CP%, digestibility of crude protein, %; GE%, digestibility of gross energy, %; EE%, digestibility of ether extract, %; CF, digestibility of crude fiber, %; NDF, digestibility of neutral detergent fiber, %.

Correlation analysis was performed between growth performance (eg., ADG, ADFI, and F/G) from 0-28d, nutrition digestibility of piglets, and long-chain fatty acid contents in the diet (Figure 3). ADG and ADFI correlated significantly and negatively with ATTD of CP, GE, EE, and NDF (*p* < 0.05). Conversely, dietary C16:0 content significantly and positively correlated with ADG and ADFI (*p* < 0.05) and significantly and negatively correlated with ATTD of EE and CF (*p* < 0.05). The levels of C18:1, C18:2, and U/S were significantly and positively correlated with ATTD of EE (*p* < 0.05) and significantly and negatively correlated with ADG and ADFI (*p* < 0.05).

### 3.7 Effect of palm oil replacement for soybean oil on microbial diversity

**Figure 4:**
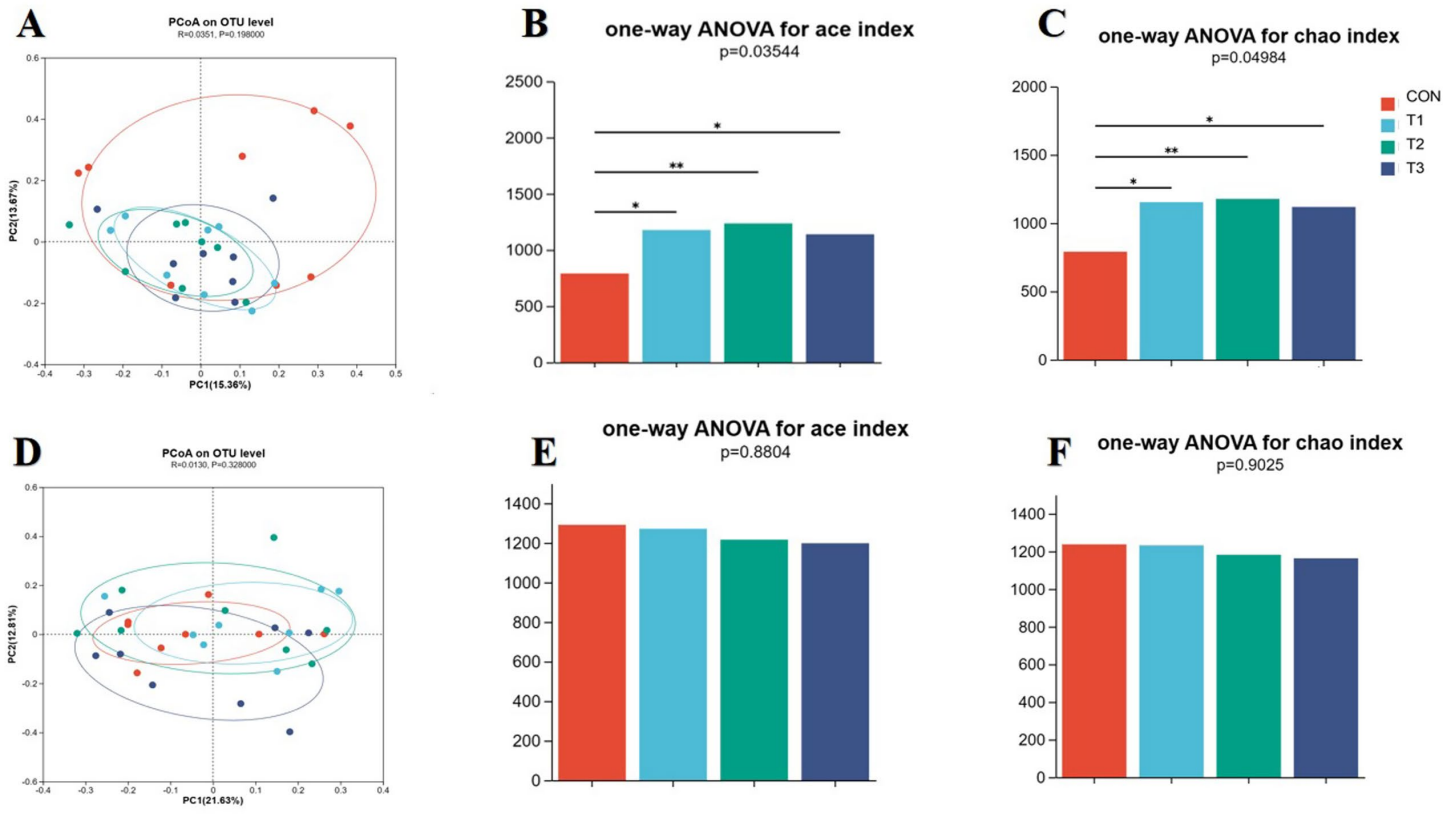
Analysis of fecal microbial diversity at 14 d and 28 d in weaned piglets. **(A)** 14-day analysis of fecal microbial PCoA. **(B)** 14-day analysis of microbial OUT abundance, ACE index, and diversity. **(C)** 14-day analysis of microbial OUT abundance, chao index, and diversity. **(D)** 14 d analysis of fecal microbial PCoA. **(E)** 28 d microbial OUT abundance ACE index diversity. **(F)** 28-day analysis of microbial OUT abundance, chao index, and diversity.

PCoA analysis revealed no significant difference in treatment diversity on days 14 (Figure 4A, *p =* 0.198) and 28 (Figure 4D, *p* = 0.328). Microbial diversity revealed the abundance of operational taxonomic units (OTU) to have increased significantly on day 14 (Figure 4B: Ace index *p* = 0.03, Figure 4C: Chao index *p* = 0.05); the abundance of operational taxonomic units (Figures 4E,F) was not significant on day 28.

**Figure 5:**
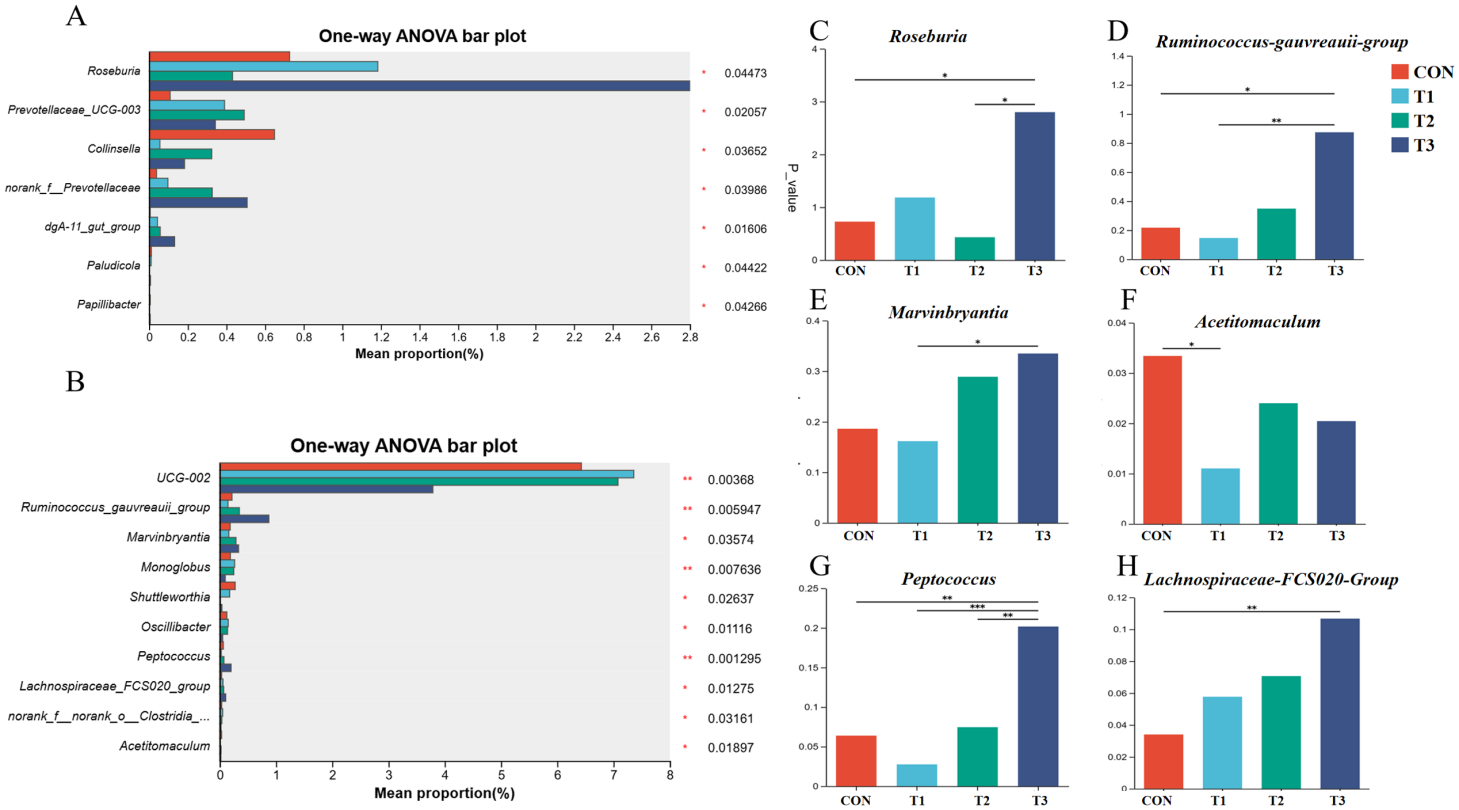
The analysis of the significance of differences between groups at the 14 d and 28 d on genus levels. **(A,B)** The top 10 abundance groups with significant differences among groups at the 14 d **(A)** and 28 d **(B)** genus levels. The Y-axis represents the species name at the taxonomic level, the X-axis represents the average relative abundance of different species groups, and different colored columns indicate different groups; on the far right is the *p*-value. **(C)** Relative abundance of *Roseburia* in the 14-d feces. **(D–H)** Relative abundance of *Ruminococcus-gauvreauii-group, Marvinbryantia, Acetitomaculum, Peptococcus,* and *Lachnospireceae-FCS020-Group* in the 28-d feces. *p < 0.05, 0.05 < **p < 0.01.

At the genus level, the relative abundance of 7 fecal microorganism genera on day 14 (Figure 5A) and 10 genera on day 28 (Figure 5B) differed significantly between treatments (*p* < 0.05). Of these, the relative abundance of *Roseburia* on 14 d in T3 was significantly higher (Figure 5C, *p* < 0.05). On day 28, the relative abundance of *Acetitomaculum* in T1 was significantly lower (Figure 5F, *p* < 0.05), and the relative abundances of *Ruminococcus-gauvreauii-group* (Figure 5D), *Peptococcus* (Figure 5G), and *Lachnospireceae_FCS020_group* (Figure 5H) in T3 were significantly higher (*p* < 0.05). The relative abundance of *Marvinbryantia* (Figure 5E) and *Peptococcus* (Figure 5G) in T3 was significantly higher than that in T1 (*p* < 0.05).

### 3.8 Relationship between fecal microbial relative abundance and growth performance

**Figure 6:**
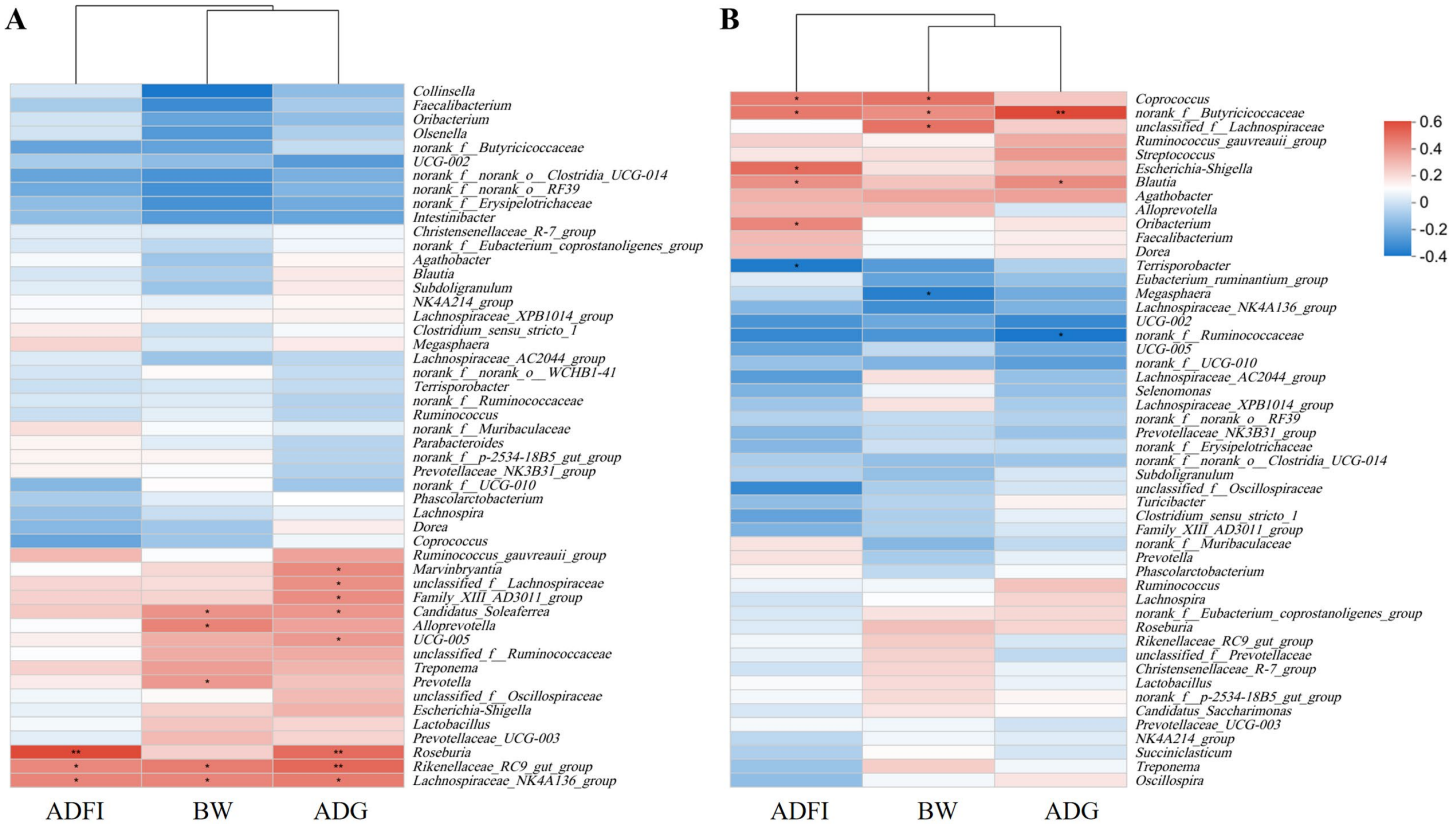
Correlation analysis of fecal microorganisms at 14 days **(A)** and 28 days **(B)** between growth performance in weaned piglets. The heat map displays the extent of the changes. The color scale ranges from saturated red (0.6) to white (0) to saturated blue (−0.4). Red and blue colors represent increased and decreased expressions, respectively. *p < 0.05, 0.05 < **p < 0.01.

The relative abundances of *Candidatus_Soleaferrea, Alloprevotella, Prevotella, Rikenellaceae_RC9_gut_group*, and *Lachnospiraceae_NK4A136_group* in feces of 14-d-old piglets correlated positively with BW; those of *UCG_005, Marvinbryantia, Ruminococcus-gauvreauii-group*, *Candidatus_Soleaferrea*, *unclassified_f__Lachnospiraceae*, *Family_XIII_AD3011_group*, *Roseburia*, *Rikenellaceae_RC9_gut_group*, and *Lachnospiraceae_NK4A136_group* correlated positively with ADG; and those of *Roseburia*, *Rikenellaceae_RC9_gut_group*, and *Lachnospiraceae_NK4A136_group* correlated positively with ADFI (Figure 6A).

On day 28, the relative abundances of *Coprococcus, norank_f_Butyricicoccaceae*, *Escherichia_Shigella*, *Blautia*, and *Oribacterium* correlated positively with BW, and that of *Terrisporobacter* correlated negatively with BW (Figure 6B). The relative abundances of *norank_f_Butyricicoccaceae*, *unclassified_f_Lachnospiraceae*, and *Coprococcus* correlated positively with ADG; the relative abundance of *Megasphaera* correlated negatively with ADG. The relative abundances of *norank_f_Butyricicoccaceae* and *Blautia* correlated positively with ADFI, and that of *norank_f_Ruminococcaceae* correlated negatively with ADFI.

## 4 Discussion

Weaning piglets often face challenges related to low feed intake and insufficient energy intake, which can hinder their growth and development. Fat supplementation can enhance energy intake in piglets (20). PO is rich in long-chain saturated fatty acids, which more effectively reduce energy expenditure and promote feed intake and weight gain than medium-chain fatty acids (6–9). The addition of 6% PO to the diet of weaned piglets significantly increased piglet ADG and ADFI (11). When PO and SO were added at 2% in a mixed fat supplement, the ADG of piglets was 18.38% higher compared with piglets that received only SO (21). Our experimental results align with these findings—that supplementing PO improves ADG and ADFI in weaned piglets. Collectively, these findings support the hypothesis that PO, because of its high content of long-chain saturated fatty acids, may stimulate appetite and improve growth performance in weaned piglets.

The hypothalamic control of feeding and energy homeostasis is mainly regulated by neurons in the arcuate nucleus, including appetitive NPY/AGRP neurons and anorexigenic pro-opiomelanocortin (POMC) neurons (22). NPY/AGRP neurons promote feeding and energy retention by increasing NPY and AGRP levels during energy-demanding states and decreasing insulin, leptin, glucose, and free fatty acids (23, 24). These neurons are important regulators of the hypothalamic appetite regulatory network, jointly participating in regulating food intake and maintaining the body’s energy balance (24–26). Palmitic acid is a long-chain saturated fatty acid, and increased expression of NPY and AGRP in hypothalamic neurons treated with palmitate can directly affect the food intake regulation pathway (27–29). Palmitic acid, the main component of a high-fat diet, directly increased NPY and AGRP mRNA levels, produced orexigenic stimuli, and led to weight gain (28, 30, 31). We report PO to increase the expression of NPY and AGRP genes in the piglet hypothalamus to correlate the ratio of C16:00 in the diet with ADFI and to simultaneously decrease serum GLU and NEFA levels. This indicates that PO promotes the expression of orexigenic genes because of its high palmitic acid content, thereby regulating piglet feeding and improving their growth performance. The positive effects of PO observed across multiple studies highlight its potential as an effective dietary supplement to optimize nutrition for weaning piglets.

The digestion and absorption of dietary oils are greatly influenced by fatty acid composition, and the U/S ratio is one of the main factors that affects oil digestibility. Li et al. (32) observed that when 10% of various oils—PO, poultry fat, fish oil, corn oil, and linseed oil—were added to diets, PO exhibited lower digestibility, metabolic energy, and net energy compared to the other oils. It was found that feeding PO also reduced the digestible energy, metabolic energy (DE and ME), and the digestibility of crude fat compared with SO (33–35). Feeding PO (U/S = 1.0) as opposed to SO (U/S = 5.7) reduces the digestible energy, metabolic energy (digestible and metabolizable), and digestibility of crude fat. The standardized ileal digestibility of dietary fat correlated positively with U/S, with the optimal U/S ratio for fat digestibility being 4.14, with a minimum ratio of 2.91 (36). We report the replacement of SO to reduce EE digestibility in piglets, and that EE digestibility correlates positively with U/S, consistent with the aforementioned results. This demonstrates that the higher the saturated fatty acid content in oil, the lower the fat digestibility. Palm fat palmitic acid content is rich (69–74% of the oil) and is not conducive to digestion and nutrient absorption. When replacing SO with 25% PO, the U/S is 2.76 and digestibility is 77.22%, which represents the best substitution rate among the experimental treatments.

This study systematically elucidates the effects of replacing SO with PO on intestinal barrier function in weaned piglets. While previous studies reported that high-dose PO (6%) negatively regulated tight junction proteins through the AMPK/mTORC1 and AMPK/Sirt1/NF-κB pathways (37), our findings demonstrate that complete replacement (4% PO) significantly upregulates the expression of intestinal barrier genes ZO-1 and Claudin-1 without suppressing intestinal development. The differential effects observed in these studies may be attributed to dosage variations, where high-dose PO activates AMPK signaling pathways that negatively regulate tight junction proteins, whereas appropriate doses can enhance intestinal barrier function. Notably, our results contrast with those of He et al. (38), who found that PO reduced the jejunal V/C ratio compared to SO, while Cong et al. (39) reported that 4% PO supplementation significantly increased intestinal Claudin-2 and ZO-1 expression in broilers, collectively suggesting that PO’s effects on intestinal barrier function are dose-dependent. These findings provide important insights for optimizing lipid supplementation strategies in weaned piglet diets. The gut microbiome plays an important role in regulating host physiology and health. Microbe richness and diversity can enhance an organism’s resistance to external infections, while different types and quantities of lipid hydrolysates may variably affect microbial composition within the same intestinal segment (40). The hydrolysis products of SO and PO differ in their fatty acid profiles, potentially leading to distinct microbial responses. PO can increase the species richness of the gut microbiota (as measured by ACE and Chao 1) (34). We report that PO increases fecal microbiota ACE and Chao indices, indicating that PO fatty acid hydrolysates can improve gut microbiota diversity. *Roseburia*, a major producer of butyrate in the gut (41), contributes to the restoration of the gut microbiome and promotes colonic motility (42). We report a positive correlation between the relative abundance of *Roseburia* and ADG and FI. Therefore, this increase in *Roseburia* abundance in the gut of weaned piglets may lead to higher butyrate levels and represent a key positive effect of replacing SO with PO on animal health. The relative abundance of the *Ruminococcus-gauvreauii-group* correlates positively with BW and ADG (43). The relative abundance of *Marvinbryantia* correlates positively with intestinal epithelial cell energy metabolism and butyrate production (44). This suggests that replacing SO with PO enhances piglet growth performance by altering microbial community composition.

This study demonstrates that replacing SO with PO in weaned piglet diets improves intestinal barrier function. These findings suggest that PO has promising potential as an alternative lipid source to optimize intestinal health and growth performance in weaned piglets. Although the T3 group exhibits superior performance in terms of ADFI, intestinal barrier function, and feed intake regulation, the T1 group achieves the optimal balance between growth promotion and high digestibility. Notably, our research represents the first integrated analysis of dietary long-chain fatty acid composition, microbial community structure, and growth phenotypes (BW/ADG/ADFI). However, the specific signaling pathways through which PO influences feed intake require further investigation.

## 5 Conclusion

Replacing SO with PO at moderate levels can positively affect appetite, promote growth performance, and regulate intestinal microbial diversity in weaned piglets. However, high substitution rates may reduce nutrient digestibility. We reported that replacing SO with 25% PO is appropriate for the diet of weaned piglets.

## Data Availability

The data presented in the study are deposited in the NCBI Sequence Read Archive (SRA), Project accession number: PRJNA1028260 Direct access https://www.ncbi.nlm.nih.gov/bioproject/PRJNA1028260.
