# Supplementary material for: Effects of replacing soybean oil with palm oil on growth performance, appetite, and gut health in weaned piglets
Source: Front Vet Sci. 2026 Feb 12;12:1682514. doi: 10.3389/fvets.2025.1682514 (PMC12935641; doi:10.3389/fvets.2025.1682514)
Supplement: Supplementary file 1 [file Table_1.DOCX]

**Supplementary Table 1.** Dietary formulation and nutritional composition of weaned piglet diets (0-14d)

| **Item** | **CON** | **T1** | **T2** | **T3** |
| --- | --- | --- | --- | --- |
| Ingredient, % |  |  |  |  |
| Corn | 25.14 | 25.14 | 25.14 | 25.14 |
| Extruded corn | 15.00 | 15.00 | 15.00 | 15.00 |
| Fermented soybean meal, 50 % CP | 5.00 | 5.00 | 5.00 | 5.00 |
| Expeller soybean meal, 46 % CP | 14.00 | 14.00 | 14.00 | 14.00 |
| Extruded soybean meal | 5.50 | 5.50 | 5.50 | 5.50 |
| Rice, Broken | 10.00 | 10.00 | 10.00 | 10.00 |
| Whey powder, 3.5 % cp | 8.00 | 8.00 | 8.00 | 8.00 |
| Fish meal, 67 % cp | 5.00 | 5.00 | 5.00 | 5.00 |
| Soybean oil | 4.00 | 3.00 | 2.00 | 0.00 |
| Palm oil | 0.00 | 1.00 | 2.00 | 4.00 |
| CaHPO4 | 0.50 | 0.5 | 0.5 | 0.5 |
| CaCO3 | 0.65 | 0.65 | 0.65 | 0.65 |
| Sucrose | 2.00 | 2.00 | 2.00 | 2.00 |
| Nacl | 0.35 | 0.35 | 0.35 | 0.35 |
| Lysine | 0.75 | 0.75 | 0.75 | 0.75 |
| Threonine | 0.28 | 0.28 | 0.28 | 0.28 |
| Tryptophan | 0.08 | 0.08 | 0.08 | 0.08 |
| Methionine | 0.12 | 0.12 | 0.12 | 0.12 |
| Mineral premix^1^ | 0.13 | 0.13 | 0.13 | 0.13 |
| Glucose | 2.50 | 2.50 | 2.50 | 2.50 |
| Zinc oxide | 0.20 | 0.20 | 0.20 | 0.20 |
| Acidifier | 0.60 | 0.60 | 0.60 | 0.60 |
| Vitamin premix ^2^ | 0.05 | 0.05 | 0.05 | 0.05 |
| Choline chloride | 0.15 | 0.15 | 0.15 | 0.15 |
| Total | 100.00 | 100.00 | 100.00 | 100.00 |
| Nutritional levels (calculated), % |  |  |  |  |
| Metabolizable energy, Kcal/kg | 3569.33 | 3566.16 | 3562.98 | 3556.62 |
| Crude protein | 18.65 | 18.65 | 18.65 | 18.65 |
| Crude fiber | 1.97 | 1.97 | 1.97 | 1.97 |
| Ether extract | 7.92 | 7.94 | 7.95 | 7.98 |
| Nutritional levels (measured) , % |  |  |  |  |
| Gross energy, Kcal/kg | 4122.14 | 4089.04 | 4124.18 | 4118.45 |
| Crude protein | 19.33 | 18.74 | 19.26 | 18.76 |
| Crude fiber | 1.31 | 1.01 | 1.07 | 1.44 |
| Neutral detergent fiber | 4.05 | 4.54 | 4.96 | 4.58 |
| Ether extract | 7.27 | 7.77 | 7.87 | 7.99 |

^1^ Mineral premix per kg diet: copper, 18.0 mg (CuSO_4_.5H_2_O); iron, 130.0 mg (FeSO_4_. H_2_O); manganese, 50.0 mg (MnSO_4_); zinc, 130.0 mg (ZnSO_4_); selenium, 0.5 mg (Na_2_SeO_3_), iodine, 0.65 mg (KI).

^2^ Vitamin premix per kg diet: vitamin A, 12,000 IU; vitamin D3, 3,600 IU;, vitamin E, 150 IU; vitamin K3, 7.2 mg; vitamin B1, 3 mg; riboflavin, 10.8 mg; vitamin B6, 5.4 mg; vitamin B12, 0.06 mg; D-pantothenic acid, 36.0 mg; niacin, 60.0 mg; folic acid, 6 mg; Biotin, 0.6 mg.
